# PRDX6 regulates the H2O2 and blue light-induced APRE-19 cell apoptosis via down-regulating and interacting with RARA

**DOI:** 10.1080/19768354.2019.1592021

**Published:** 2019-04-11

**Authors:** Xu Zha, Guojiu Wu, Hong Zhang, Yanni Yang, Yuanping Zhang, Linkun Ma

**Affiliations:** Department of the Ophthalmology, Second Affiliated Hospital of Kunming Medical University, Kunming, People’s Republic of China

**Keywords:** PRDX6, RARA, APRE-19 cell, apoptosis

## Abstract

Hereditary retinal disease (HRD) is the primary retinal degeneration that leads to severe visual impairments and refractory blindness, and the therapy of HRD was most important in ophthalmology. The apoptosis of retinal cells plays important roles in HRD progression. Therefore, in this study, we explore the mechanism of H2O2 and blue light-induced apoptosis of ARPE-19 cells. Co-immunoprecipitation (Co-IP) is employed to test the interactions between proteins, and western blotting is used to detect the protein levels. Apoptosis is analyzed by Flow cytometry. Our results found that PRDX6 could interact with RARA in ARPE-19 cells, and H2O2 and blue light could significantly reduce the RARA protein expression, and also could inhibit the interaction between PRDX6 and RARA. Using a rescue experiment, we further elucidated that H2O2 and blue light reduced the RARA expression via down-regulating PRDX6. And H2O2 and blue light induced the ARPE-19 cell apoptosis via decreasing the expression of PRDX6. Our results suggested that the interaction between PRDX6 and RARA played important roles in the apoptosis of ARPE-19 cells.

## Introduction

Hereditary retinal disease (HRD) was the primary retinal degeneration which causes visual impairments and refractory blindness (Goodwin 2008). HRD has the highest incidence, and in China, the average incidence was about 1/1000 (Wright et al. 2010). Therefore, exploring the novel therapy for HRD was urgently needed.

The retinal pigment epithelium (RPE) was involved in maintaining photoreceptor structure and role. However, the cells of RPE were susceptible to oxidative stress including H2O2 and blue light. In vitro, oxidative stress caused severe damage to the cells of RPE, and resulted in photoreceptor cell apoptosis, which leads to the development of HRD (Vlachantoni et al. 2011). Meanwhile, in vivo, HRD mice had the vulnerability to short-wavelength radiation because of higher reactive oxygen species levels (Ho et al. 2015).

PRDX6 belongs to the peroxiredoxin family which was reported as the enzymes implicated in the regulation of H2O2 signaling and the protection from oxidation (Ambruso 2013). Like other PRDXs, PRDX6 could decrease the short-chain hydroperoxides by using its peroxidase activity. The PLA2 activity specific to PRDX6 was reported to have both antioxidant properties and pro-oxidant properties (Chatterjee et al. 2011; Lien et al. 2012).

PRDX6 was reported to play important roles in the pathogenesis of abdominal aortic aneurysm (Burillo et al. 2016). Choi et al. found that PRDX6 was significantly decreased in the rat cerebral ischemic injury model with human cerebral endothelial cell transplantation (Choi et al. 2016). Sahu et al. reported that PRDX6 overexpression was associated with poor prognosis of several types of cancers, and miR-371-3p played important roles in leading to drug tolerance via targeting PRDX6 and regulating PLA2/PKCα activity (Sahu et al. 2016). Singh et al. reported that PRDX6 was decreased in the neuronal cells under stress, and the ROS level was increased, and the cells subsequently underwent apoptosis. And transduction of PRDX6 conferred resistance to the oxidative stress causing by paraquat, H2O2, and glutamate (Singh et al. 2016). Although PRDX6 plays an important role in several disease progressions, the role and mechanism of PRDX6 in HRD are still largely unknown.

Our previous study found that H2O2 and blue light could induce oxidative stress and cell death via decreasing PRDX6 and inhibiting PI3K/AKT in ARPE-19 cells (Zha et al. 2015). In the present study, we further explored the mechanism of cell death induced by H2O2 and blue light, and revealed that interaction of PRDX6 and RARA played an important role in H2O2 and blue light-induced apoptosis.

## Method

### Cell culture

The human retinal pigment epithelial cell line (ARPE-19) was purchased from American Type Culture Collection (ATCC, USA), and was cultured in Dulbecco’s modified Eagle’s medium (DMEM) supplemented with 10% fetal bovine serum (FBS), at 37°C and 5% CO_2_.

### Cell transfection

Cells were transfected with the plasmids of pcDNA3.1-PRDX6 or PRDX6-shRNA using Lipofectamine 2000 (Invitrogen, USA) in serum-free DMEM according to the manufacturer's instructions. After 48 h, the transfected cells were harvested.

### Apoptosis detection

Apoptosis was analyzed using the Annexin V-FITC Apoptosis Detection Kit (Beyotime, Nantong, China) according to the manufacturer's protocol. ARPE-19 cells were seeded in the 6-well plates at a density of 4 × 10^6^ cells/ml. At 48 h after drug treatment, cells were harvested, and analyzed using a flow cytometer (FACS Partec, Germany) according to the manufacturer's protocol.

### Western blot analysis

Cells were isolated using the protein lysis buffer (Beyotime, China) according to the manufacturer’s instructions. The primary antibodies used in our study were an anti-PRDX6 antibody (abcam, 1:1000), anti-RARA antibody (abcam, 1:1000), anti-β-Actin antibody (abcam, 1:2000) overnight at 4°C. β-Actin was used as the reference control.

### Co-immunoprecipitation

Total proteins of ARPE-19 cells were extracted using IP lysis buffer (Thermo Scientific, USA). PRDX6 antibody (Abcam) or control immunoglobulin (IgG) (Cell Signaling, USA) were incubated with cell lysate overnight at 4°C. Then followed by protein A Dynabeads (Invitrogen, USA) incubation for 2 h at 4°C. After washing for three times, the pulled-down proteins were examined by Western blotting analysis.

### Statistical analysis

All data were analyzed using two-tailed Student’s t test and expressed as mean ± SD between two groups. Significance was set at *p* < .05.

## Results

### H2O2 and blue light reduced the expressions of PRDX6 and RARA and inhibited the interaction between PRDX6 and RARA in ARPE-19 cells

**Figure 1.**
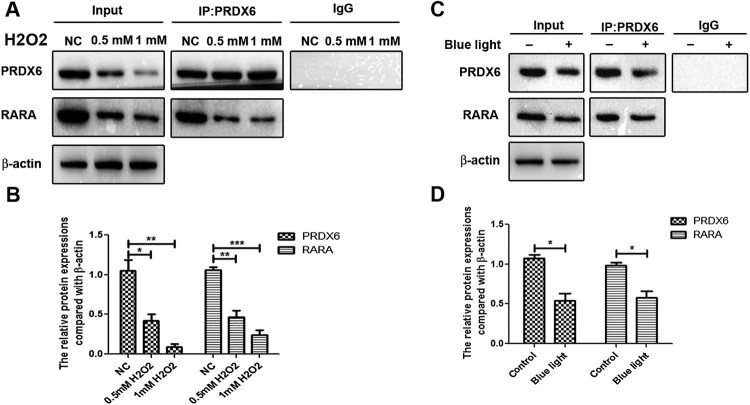
H2O2 and Blue light down-regulated PRDX6 and RARA expressions, and inhibited the interaction between PRDX6 and RARA in ARPE-19 cells respectively. (A) Co-IP analysis of the interaction between PRDX6 and RARA. (B) Statistical analysis of Co-IP results of A. (C) Co-IP analysis of the interaction of PRDX6 and RARA. (D) Statistical analysis of Co-IP results of C. *P < .05; **P < .01; ***P < .001.

We analyzed the effects of H2O2 and blue light on the ARPE-19 cells. The results found that H2O2 could significantly reduce the protein expressions of PRDX6 and RARA in a dose dependent manner (Figure 1(A,B)). Co-IP assay further found that PRDX4 could interact with RARA, and H2O2 could inhibit the interaction of PRDX6 and RARA in ARPE-19 cells.

Blue light significantly decreased the protein expressions of PRDX6 and RARA in ARPE-19 cells (Figure 1(C,D)). Co-IP assay further found that PRDX6 could interact with RARA, and blue light could inhibit the interaction of PRDX6 and RARA in ARPE-19 cells.

### PRDX6 positively regulated the protein expression of RARA under the conditions of H2O2 and blue light

In order to further study the relationship between PRDX6 and RARA, we conducted the pcDNA3.1-PRDX6 to overexpress PRDX6 and used PRDX6-shRNA to silence the expression of PRDX6 in ARPE-19 cells (data now shown).

**Figure 2.**
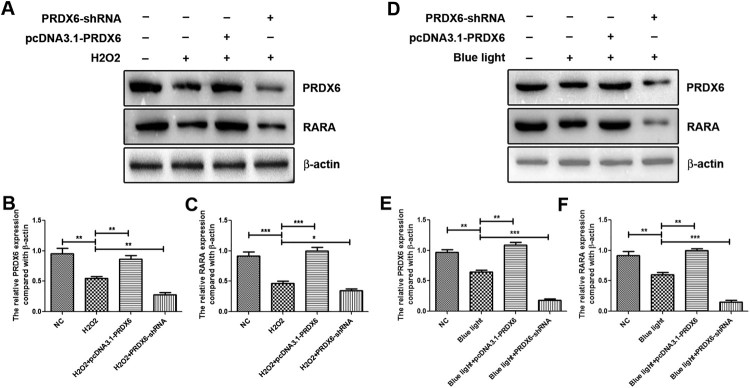
PRDX6 positively regulated RARA expression under H2O2 and blue light treatment respectively. (A) Western blotting analysis of PRDX6 and RARA. (B and C) Statistical analysis of Western blotting results of A. (D) Western blotting analysis of PRDX6 and RARA. (E and F) Statistical analysis of Western blotting results of D. *P < .05; **P < .01; ***P < .001.

H2O2 reduced the PRDX6 and RARA expressions, and overexpression of PRDX6 restored the RARA expression under H2O2 treatment, and silencing of PRDX6 further decreased the RARA expression comparing with H2O2 treatment (Figure 2(A–C)). In the condition of blue light, the expression levels of PRDX6 and RARA were decreased, and overexpression of PRDX6 could rescue the down-regulation of RARA under blue light treatment, and inhibition of PRDX6 could further down-regulate the RARA expression comparing with H2O2 treatment (Figure 2(D–F)).

### H2O2 and blue light-induced apoptosis via down-regulating PRDX6 in ARPE-19 cells

**Figure 3.**
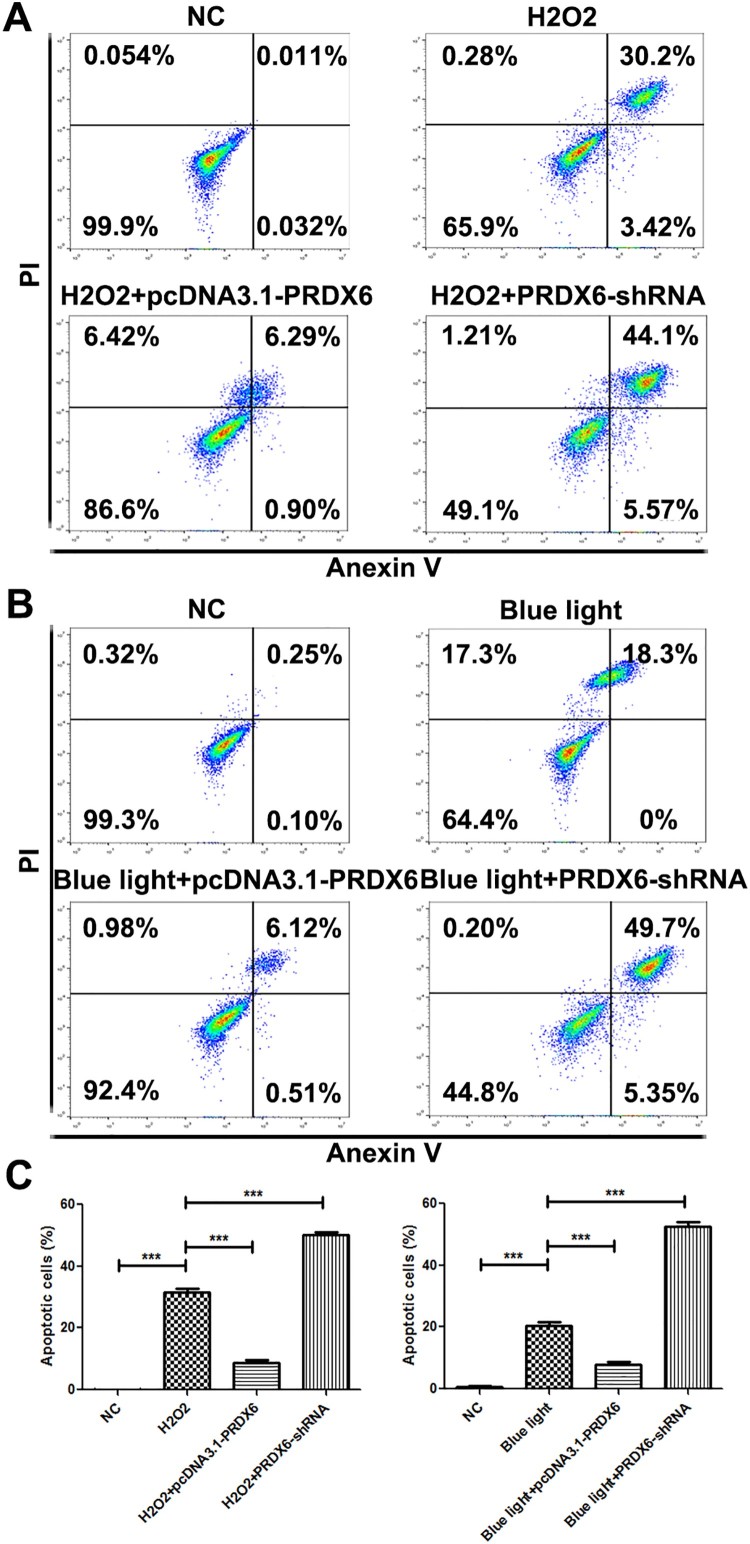
H2O2 and blue light promoted apoptosis of ARPE-19 cells via reducing PRDX6. (A) Flow cytometry analysis of ARPE-19 cells under the conditions of NC, H2O2, H2O2 + pcDNA3.1-PRDX6, H2O2 + PRDX6-shRNA. (B) Flow cytometry analysis of ARPE-19 cells under the conditions of NC, blue light, blue light + pcDNA3.1-PRDX6, blue light + PRDX6-shRNA. (C) Statistically analysis of flow cytometry data. *P < .05; **P < .01; ***P < .001.

H2O2 and blue light significantly promoted the cell apoptosis (Figure 3(A–C)). Overexpression of PRDX6 could inhibit the apoptosis induced by H2O2 and blue light (Figure 3(A–C)). Silencing of PRDX6 could further promote the apoptosis than H2O2 or blue light treatment only (Figure 3(A–C)).

## Discussion

Many studies reported that excessive light exposure could lead to a photochemical reaction in the retina, and resulted in the injury of RPE cells and the neural retina (Hadziahmetovic et al. 2012; Hunter et al. 2012). Macular degeneration and other retinal degenerative diseases were associated with the light-induced RPE damage (Xu et al. 2010). However, the mechanism of the apoptosis of RPE cells is still largely unknown.

Many studies have explored the roles of PRDX6 in cell apoptosis. Anwar et al. reported that the mRNA and protein expression levels of PRDX6 were elevated in canine haemangiosarcoma, and silencing of PRDX6 significantly promoted apoptosis of canine haemangiosarcoma cells (Anwar et al. 2016). The level of PRDX6 was lower in hepatocellular carcinoma, and its lower expression was an independent risk factor of poor prognosis. When the cancer cells were treated with H2O2, PRDX6 inhibited the apoptosis, however when treated with TNF-α, PRDX6 promoted the cell apoptosis (Xu et al. 2016). Our previous study found that PRDX6 protected ARPE-19 cells from H2O2-induced oxidative stress and apoptosis and that this protection was mediated at least partially by the PI3K/AKT pathway. However, the mechanisms of PRDX6 in H2O2 and blue light-induced ARPE-19 cell apoptosis are still largely unknown. Our results showed that PRDX6 could interact with RARA in ARPE-19 cells, and H2O2 and blue light could significantly reduce the RARA protein expression, and could also inhibit the interaction between PRDX6 and RARA. Our results suggested that the interaction of PRDX6 and RARA played important roles in ARPE-19 cell apoptosis. Using a rescue experiment, we further elucidated that H2O2 and blue light reduced the RARA expression via down-regulating PRDX6. And H2O2 and blue light induced the ARPE-19 cell apoptosis via decreasing the expression of PRDX6.

Taken together, our results firstly found that PRDX6 could interact with RARA and reduce the expression of RARA. PRDX6 regulates the H2O2 and blue light-induced ARPE-19 cell apoptosis. Future studies should focus on the mechanisms of PRDX6 and RARA, and its roles in the progression of eye diseases.
